# Editorial: Advances in nanomedicine: revolutionizing healthcare with nanoscale innovations

**DOI:** 10.3389/fbioe.2025.1736031

**Published:** 2025-11-11

**Authors:** Kexiao Yu

**Affiliations:** Department of Orthopedics, Chongqing Traditional Chinese Medicine Hospital, The First Affiliated Hospital of Chongqing University of Chinese Medicine, Chongqing, China

**Keywords:** nanomedicine, healthcare, nanoscale, advances, biotechniques

At the forefront of biomedical research, nanomedicine has rapidly evolved from a promising concept into a tangible transformative force. By engineering materials that interact with biological systems at the nanoscale, it offers unprecedented precision and multi-functionality for tackling some of the most intractable healthcare challenges. This Research Topic, “*Advances in Nanomedicine: Revolutionizing Healthcare with Nanoscale Innovations*,” showcases the latest progress in this dynamic field. The collected articles not only highlight the enhanced capabilities of nanotechnology in diagnosis and therapy but also profoundly illustrate its potential to drive the development of theranostics and precision medicine.

A central theme emerging from this Research Topic is the exploitation of the inherent biological functions of nanomaterials to transcend their traditional role as mere drug delivery vehicles. A prime example is the iron-capsaicin nanozyme developed by Wang et al. Their work moves beyond the conventional delivery of anti-inflammatory drugs; instead, the nanozyme itself, leveraging its enzyme-mimicking properties, actively remodels the inflammatory microenvironment in sepsis-induced acute lung injury by regulating macrophage polarization and scavenging reactive oxygen species. This strategy of “active regulation” over “passive transport” marks a significant evolution in the design philosophy of nanomedicines.

Similarly, in the realm of pain management, the work by Qiao et al. and Song et al. collectively expands the scope of analgesic nanomedicines from “sustained release” to “intelligent responsiveness and intrinsic therapy.” Qiao et al. constructed an intrinsic anti-inflammatory nanomedicine, where the liposomal coating composed of ginsenoside Rg3 not only facilitates sustained release but also intrinsically inhibits macrophage activation. This approach simultaneously delivers the local anesthetic levobupivacaine and directly intervenes in inflammation, a key driver of pain. Song et al. advanced this further with an ultrasound-responsive phase-transitional nanoplatform, enabling on-demand and intensity-tunable postoperative analgesia. This platform integrates diagnostic ultrasound imaging with therapeutic drug release, allowing clinicians to visualize drug distribution and precisely control analgesic intensity based on a patient’s real-time feedback, charting a course for personalized treatment.

Achieving precise delivery to specific subcellular organelles represents a higher goal of nanomedicine. In this regard, the mini-review by Buck et al. provides a succinct and insightful overview of mitochondria-targeted nanoparticles for treating brain disorders. The review emphasizes that interventions targeting mitochondria, the cellular powerhouses, offer novel hope for treating conditions like Parkinson’s disease that are closely linked to mitochondrial dysfunction. It compellingly argues that engineered mitochondria-targeted nanoparticles can achieve superior efficacy at lower drug doses and restore mitochondrial dynamics compared to their non-targeted counterparts, providing clear direction for developing next-generation neurotherapeutics.

Finally, the comprehensive review by Wang et al. offers a broad perspective, systematically elaborating on the utilization of nanomaterials in MRI contrast agents and their role in image-guided therapy. From superparamagnetic iron oxide nanoparticles to quantum dots, the article details how various nano-contrast agents improve diagnostic accuracy by enhancing imaging contrast and serve as multifunctional platforms integrating targeted drug delivery, hyperthermia, radiation therapy, and immunotherapy. This review makes it abundantly clear that nanotechnology is the core engine propelling modern medicine toward an era of visualizable, quantifiable, and regulatable theranostics.

Collectively, the contributions in this Research Topic demonstrate that nanomedicine is transitioning from a simple “carrier” role into an “intelligent system” capable of sensing, responding to, and regulating complex pathological microenvironments. These studies collectively point toward a future where medical interventions will become more precise, personalized, and controllable, with minimized side effects. Nevertheless, the path to widespread clinical translation faces shared challenges, including long-term biocompatibility assessment, standardization for large-scale manufacturing, and regulatory pathways. We are confident that through sustained and close collaboration between materials scientists, engineers, and clinicians, these hurdles will be overcome. The innovative work presented in this Research Topic represents a solid step toward this promising future, not only reflecting the cutting edge of current research but also inspiring new ideas to continue driving the nanomedicine revolution forward.

